# A Hypnic Hypothesis of Alzheimer's Disease

**DOI:** 10.1159/000350060

**Published:** 2013-04-26

**Authors:** Camilla N. Clark, Jason D. Warren

**Affiliations:** Dementia Research Centre, UCL Institute of Neurology, University College London, London, UK

**Keywords:** Sleep, Alzheimer's disease, Neurodegeneration, Default mode network

## Abstract

**Background:**

Understanding the pathophysiology of Alzheimer's disease (AD) is of fundamental importance for improved diagnosis, monitoring and ultimately, treatment.

**Objective:**

A role for the sleep-wake cycle in the pathogenesis of AD has been proposed, but remains to be worked out in detail.

**Methods:**

Here we draw together several lines of previous work to outline a ‘hypnic hypothesis’ of AD.

**Results:**

We propose that altered function of brainstem neurotransmitter pathways associated with sleep, promotes regionally specific disintegration of a cortico-subcortical ‘default mode’ brain network that is selectively vulnerable in AD.

**Conclusion:**

The formation of a dynamic toxic state within this vulnerable network linked to sleep-wake disruption, would in turn lead to failure of synaptic repair, increased transmission of pathogenic misfolded proteins and a self-amplifying neurodegenerative process. We consider the evidence for this hypnic hypothesis and the implications that follow on from it.

## Introduction

Understanding the pathophysiology of Alzheimer's disease (AD) is of fundamental importance for improved diagnosis, monitoring and ultimately, treatment of this devastating condition. Molecular biological, biochemical and neuroimaging studies have yielded a substantial body of data on the causes and evolution of AD [1,2,3,4,5], whilst the concept of a vulnerable distributed brain network provides a framework for explaining how molecular events might scale up to a clinical phenotype [6,7,8,9]. It is increasingly clear that AD has a protracted prodromal phase prior to clinical onset during which potentially pathogenic factors could operate and might have cumulative effects [5].

On a biochemical level, synaptic alterations are hypothesised to play a key role in the pathogenesis of many neurodegenerative diseases, including AD [9,10,11]. Such alterations may promote the transfer of pathogenic molecular species (in particular, β-amyloid oligomers) leading to the diffusive spread of misfolded proteins (in particular, hyperphosphorylated tau) throughout a vulnerable, distributed brain network, the so-called ‘default mode network’ (DMN) that appears to be integral to the operation of the normal resting brain [12,13,14,15,16]. Most accounts of synaptic dysfunction in AD emphasise molecular and biochemical events impacting on synaptic physiology via a loss of structural integrity [11,17]. However, an alternative (and by no means mutually exclusive) possibility is the promotion of synaptic damage by altered patterns of synaptic activity and excitotoxicity [18]. A fundamental example of a pervasive, phasic alteration in synaptic activity is the circadian sleep-wake cycle, which is associated with profound changes in many aspects of cellular and synaptic function [19,20].

The precise implications of sleep-wake alterations for the pathogenesis of neurodegenerative diseases such as AD remain to be defined. However, converging evidence drawn from molecular biology, sleep neuroscience [21,22,23,24,25,26,27,28,29,30,31,32,33,34] and clinical neurology [35,36,37,38,39,40,41,42] suggests that sleep may interact in diverse and important ways with the pathophysiology of AD. Ramifications of long-term disruption in the sleep-wake cycle could include synaptic overactivity and excitotoxicity [10,21,43,44,45], impaired synaptic pruning [46], failure of synaptic scaling and homeostatic responses [19,20,27,47,48,49,50,51], transmission of pathogenic proteins (β-amyloid and tau) [9,15,16], and disruption of neural circuitry in the vulnerable DMN [6,8,12,21,52,53]. Such alterations might in turn underpin or contribute to the cognitive syndrome that characterises AD.

## A ‘Hypnic Hypothesis’ of AD

**Fig. 1:**
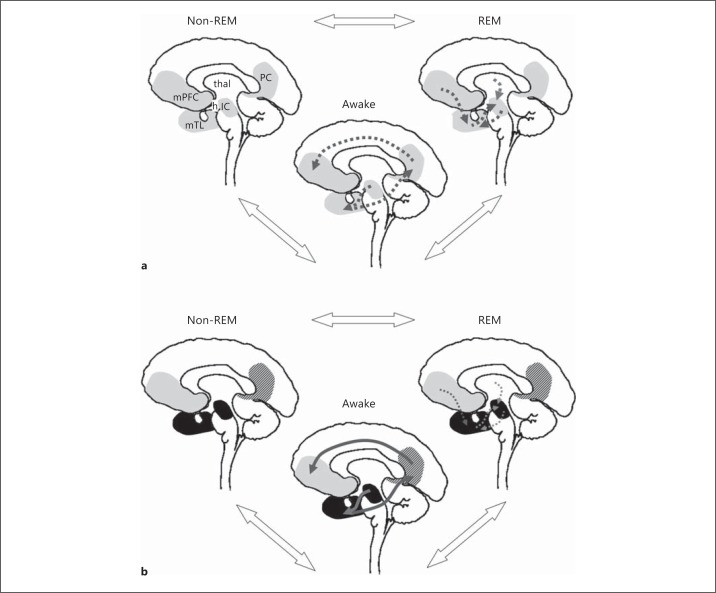
A schematic representation of the hypnic hypothesis of AD pathophysiology. The panels show non-sleep-deprived (**a**) and sleep-deprived (**b**) states. The circadian cycle is shown in simplified form comprising awake, REM sleep and non-REM sleep phases (state transitions shown as bidirectional, open arrows). The brain in each state is represented in a stylised mid-sagittal section. **a** Non-sleep-deprived state. Key components of the DMN and its subcortical projections implicated in AD are shown in grey; other areas playing a critical role in sleep physiology and linked to DMN are also shown. IC = Isodendritic core in the upper brainstem; mTL = medial temporal lobe; mPFC = medial prefrontal cortex (here including anterior cingulate); PC = posterior cingulate/precuneus; h = hypothalamus; thal = thalamus. According to the hypnic hypothesis of AD, the non-sleep-deprived state is associated with an equilibrium between processes associated with increased synaptic activity that promote neurodegeneration during waking and counteractive processes during the relatively tonically quiescent phase of non-REM sleep and an active ‘rescue’ phase of synaptic remodelling associated with REM sleep. Cumulative synaptic dysfunction associated with waking directs pro-inflammatory and pro-fibrillogenic alterations in anatomically restricted projection zones in the DMN (dotted arrows); these alterations are actively opposed especially during REM sleep (reversed dotted arrows). **b** Sleep-deprived state. In this state the net balance of effects favours neurodegeneration, and repair processes associated with sleep phases are inefficient or attenuated, with consolidation of neuronal damage leading ultimately to chronic neuronal dysfunction (cross-hatched grey areas) and frank neuronal loss (black areas). The topography of brainstem projections and inter-linked DMN regions gives rise to a specific anatomical evolution of AD pathology targeting the DMN (solid grey arrows). AD itself induces sleep disruption due to early involvement of IC, leading to a toxic, self-amplifying and self-perpetuating state of chronic sleep disruption. Not shown explicitly in the figure is the spectrum of factors (including neurotransmitter and melatonin shifts and reactive oxygen and inflammatory intermediates) associated with sleep disruption that further amplify and perpetuate the neurodegenerative process (see text for details).

Although a role for the sleep-wake cycle in the pathogenesis of AD has been proposed previously [26,33,45,54], this idea has received relatively little attention; however, it has become increasingly compelling in light of recent findings in human molecular and neuroimaging studies. Here we draw together several lines of previous work [1,2,8,10,12,13,14,21,33,35,36,37,39,42,43,55,56,57,58,59,60,61,62,63,64,65,66,67] to outline a hypnic hypothesis of AD pathophysiology. A prima facie case for the hypothesis rests on accumulating evidence that sleep-wake cycle disturbances are significant in AD, and may track disease evolution [35,36,37,39,55,58,61,62,67]. The key features of the hypothesis we propose and predictions that follow from it are outlined in table 1 and figure 1.

Ascending neurotransmitter projection pathways play a key role in initiating and maintaining the brain state shifts that underpin sleep and wakefulness [68]. Aminergic and cholinergic pathways originating in brainstem nuclei govern thalamocortical activity changes that in turn evoke the cortical activity profile of physiological sleep stages [59,64]. During sleep, these ascending transmitter systems modulate the activity of key component areas within the DMN [12,13,64]. The DMN includes the mesial temporal lobes and outflow projections to the posterior cingulate, to the medial and inferior parietal and prefrontal cortices and their subcortical connections [7,12]. The core function of the network continues to be defined, but it is active during stimulus-independent thought in the resting wakeful (and dreaming) brain [7,59,60]. Functional alterations in the DMN have been consistently identified in neuroimaging studies [6,7,8] of AD. Brainstem systems projecting to the DMN together with the DMN proper are likely to be integral to the development of AD; brainstem nuclei (in particular, the locus coeruleus) and ascending neurotransmitter systems comprise the ‘isodendritic core’ or reticular formation [66] and are among the earliest targets of AD pathology [1].

Emerging evidence suggests a fundamental, functional linkage between the neurobiology of sleep and AD. Sleep, in particular rapid eye movement (REM) sleep, is postulated to have restorative effects on neuronal function [67] and may promote synaptic efficiency [21,46]. The role played by this process has recently been emphasised as a guiding principle of brain operation in a novel account of sleep neurobiology proposed by Hobson and Friston [46]. According to this account, ascending brainstem neurotransmitter systems interacting with components of the DMN are essential for maintaining and updating the brain's internal model of the world by minimising redundant complexity in synaptic networks. Such a generic sleep-related function would provide a mechanism linking increased time awake due to sleep deprivation with inefficient synaptic firing and increased vulnerability of neural networks hosting these dysfunctional synaptic connections. As a core projection target of the neurotransmitter pathways that mediate sleep-wake cycling, the DMN may be particularly susceptible to the effects of sleep disruption. Such selective susceptibility of the DMN is predicted by the hub vulnerability theory, according to which synaptic overactivity facilitates neurodegeneration [21,43]. Synaptic effects of sleep deprivation are unlikely to be restricted to a single pathophysiological mechanism; for example, synaptic overactivity, inefficient pruning or remodelling of redundant synapses and perturbed synaptic scaling and homeostasis might all operate within the DMN over different timescales and would be mutually reinforcing.

While sleep promotes complexity reduction and the regulation of redundant synaptic connections, ongoing involvement of the DMN in these processes during wakefulness is very likely on anatomical grounds. If correct, this account would therefore place the DMN and its modulation by sleep at the foreground of brain function across the circadian cycle both in health and in AD. A disease state such as AD that initially damages sleep control mechanisms in the isodendritic core would have potentially widespread and cumulative effects on synaptic function in the core and its projection zones in the DMN [66]. In particular, if the dynamic toxic state generated within the network and the associated failure of synaptic repair lead to accumulation and transmission of pathogenic misfolded proteins, then neural damage would in time become self-amplifying and self-perpetuating (see fig. 1). [10,21,24].

We now consider the evidence for the hypothesis and its implications in more detail.

## Evidence for the Hypothesis: Sleep Alterations in AD

A hypnic hypothesis of AD requires, firstly, that sleep disturbance should form part of the early clinical phenotype. Indeed, though difficult to study rigorously outside specialised sleep laboratories, substantial evidence suggests that sleep-wake cycle disruption is a significant issue detectable in early AD and becomes more severe with disease evolution. It has been proposed that chronic sleep deprivation and associated neuronal overactivity may directly confer an increased risk of developing AD [26]. Sleep disturbance is more frequent in patients with AD than the general population [36], occurring in some 25-54% of patients overall [54,58]; as this is based largely on caregiver reports, it may be an underestimate. Sleep disturbance occurs early in the course of disease [61] and worsens as disease evolves [27,69], though not necessarily in a simple monotonic relation [39,63]. Sleep disruption is an important determinant of institutionalisation in later-stage disease [42]. Normal ageing is associated with increased sleep latency and frequent night time awakenings [24] leading to increased daytime sleepiness [36,37], features which are exaggerated in AD [39]. AD is associated with decreased slow-wave sleep and REM sleep, prolonged REM latency, increased proportions of stages I and II sleep, and increased fragmentation of sleep, leading to an overall decrease in sleep duration [36,39]. Even after accounting for the effects of increased sleep fragmentation, AD is also associated with shifts in the normal circadian alertness profile (contributing to ‘sundowning’ in later-stage disease) [35,36,37], consistent with a specific impairment of circadian pacemaker function.

Very little information is currently available concerning dream morphology in AD. Nightmares can be a prominent feature of treatment with cholinesterase inhibitors, although the mechanism is debated [70,71]. Alteration in several sleep parameters correlates with measures of performance across cognitive domains in AD [37,39], suggesting a pervasive effect on cognitive function.

An obvious question in this situation is whether the correlation of increased cognitive dysfunction and sleep disruption in AD might simply reflect the known cognitive effects of sleep deprivation. This seems unlikely as the cognitive effects of sleep deprivation are attenuated during normal ageing [69], while the cognitive parameters linked to sleep disturbance in AD mirror the typical cognitive profile of the disease [37,39]. More direct evidence linking sleep disturbance with subsequent neurodegeneration has been obtained in animal models; alterations of REM sleep and circadian architecture similar to those in human AD patients precede deposition of amyloid plaques in APP transgenic mice [25].

## Evidence for the Hypothesis: The Neurobiology of Sleep

A second line of evidence linking sleep pathophysiology with development of AD pathology rests on the common involvement of brainstem neurotransmitter systems in these processes. The normal sleep-wake cycle is governed by the interaction of neurochemical (in particular, cholinergic and aminergic) systems in the upper brainstem, hypothalamus and basal forebrain and their subcortical and cortical projections. The cholinergic forebrain innervation originates mainly in the basal nucleus of Meynert and the noradrenergic innervation in the locus coeruleus [63]. Together with orexin released by the posterolateral hypothalamus, the ascending pathways diffusely promote wakefulness; GABA- and galanin-mediated inhibition of these pathways by the rostral hypothalamus triggers sleep onset, while mutual inhibition between pathways regulates transitions among sleep stages [68]. The circuitry mediating sleep-wake transitions is modulated by signals sent from the circadian pacemaker in the suprachiasmatic nucleus and by humoral factors (in particular, melatonin), allowing entrainment with environmental factors such as the day-night cycle [68]. The neuronal systems mediating the circadian cycle project to areas within the DMN [63,66,72,73].

The stages of sleep and wakefulness have characteristic electrophysiological signatures, reflecting underlying thalamocortical activity patterns: non-REM sleep is associated with slow delta and ‘spindle’ activity due to oscillatory firing of thalamic neurons and synchronised cortical activity, while wakefulness and REM sleep are associated with thalamocortical desynchronisation [46,64,68]. Functionally, non-REM sleep is likely to be crucial for memory consolidation and synaptic regulation more generally [74], while REM sleep may serve to develop and maintain normal waking consciousness by building an internal predictive ‘model’ of the world that can then be tested and refined in response to waking sensory experience [46,65]. The noradrenergic locus coeruleus fires maximally during wakeful states, decreases its activity during non-REM sleep and is silent during REM sleep [68]. The serotonergic dorsal raphe nucleus also fires maximally during wakefulness and suppresses REM sleep [68]. Cholinergic neurons in the pons and basal forebrain fire during wakefulness and REM sleep [54]. Acting together via their specific effects on sleep stage transitions, multiple parallel ascending neurotransmitter systems modulate behavioural arousal, allowing programming of flexible motor and affective responses to the internal milieu and the external environment [68]. Endogenous circadian pacemaker effects on cognition and behaviour interact with (but are partly separable from) the effects of elapsed wakefulness and sleep periods per se [75]. This complex neurochemical milieu would also provide substrates to link sleep-wake alterations with mood alterations and AD [40,42].

In human functional neuroimaging studies, both non-REM and REM sleep are associated with complex, phasic, regionally specific profiles of cortical activity changes [51,59]. Non-REM sleep is associated with tonic deactivation of brainstem, thalami, cingulate, mesial temporal and prefrontal areas; however, non-REM slow-wave activity is associated with increased activation of brainstem, ventral prefrontal, posterior cingulate/precuneus and parahippocampal areas [51,59]. Sleep spindles are associated with additional activation of thalamus, hippocampus, anterior cingulate and sensorimotor areas [74,76]. REM sleep is associated with activation of brainstem, thalami, anterior cingulate, mesial temporal and paralimbic areas and deactivation of posterior cingulate/precuneus, parietal and inferior frontal areas [51,59,60,77]. These activation profiles overlap extensively with the DMN, but also show that activity shifts are not uniform across the network [60]. Although the core function of the DMN remains controversial [78], it is known to mediate stimulus-independent thought during wakefulness. Activation of the DMN during REM sleep is therefore plausible a priori, since dreaming is arguably the purest example of stimulus-independent thought [46]. REM sleep is associated with specific alterations in DMN connectivity (in particular, reduced connectivity between posterior cingulate and more anterior regions) and reduced reactivity to sensory stimuli, which together may mediate REM-specific functions linked to ‘isolation’ of DMN subsystems from the external environment [59,79].

Wakefulness tends to promote a net increase in synaptic strength, while sleep tends to attenuate synaptic strength [20,80,81], which is consistent with the known restorative and homeostatic functions of sleep. However, these sleep functions are unlikely to reflect the net direction of activity shifts alone. For example, wakefulness and REM sleep are associated with similar metabolic profiles, suggesting that the quality (and not merely the overall amount) of synaptic activity mediates the restorative functions of the REM state. One potential expression of this qualitative alteration in synaptic drive may be a redistribution of connectivity between components of the DMN [79]. Although slow-wave (non-REM) sleep has generally been emphasised in synaptic homeostasis and downscaling of synaptic networks potentiated during wakefulness, recent evidence suggests that REM sleep plays an important role in these processes [47]. In particular, REM sleep is associated electrophysiologically with a specific profile of phasic synchrony shifts in hippocampal subregions [29], providing a substrate for altered network synchrony, synaptic plasticity and information transfer which may be regionally specific [52]. REM sleep deprivation in rats blocks expression of brain-derived neurotrophic factor [48], a key signalling molecule that modulates neural circuit activity and synaptic strength [18], while modafinil ameliorates the cognitive effects of REM deprivation in mice via increased expression of MMP-9, a gene implicated in synaptic plasticity [34]. We suggest that REM sleep may serve a crucial and active ‘rescue’ function in the repair of incipient neurodegenerative changes within synaptic circuits concentrated in the DMN.

REM sleep disruption early in the clinical course of AD is likely in part to result from degeneration of ascending cholinergic pathways, as these pathways are critical for initiating and maintaining REM sleep [54]. Chronic sleep deprivation in rats has been associated with the formation of anti-nerve growth factor antibodies [22] which, when infused into rat cerebral cortex, result in neurodegeneration of cortical cholinergic boutons [82], and transgenic anti-NGF mice develop Alzheimer-like neurodegeneration [83], suggesting a possible mechanism for sustained deleterious effects. The acetylcholinesterase inhibitor, donepezil, restores REM sleep in patients with mild-to-moderate AD, and the magnitude of this effect correlates with cognitive benefit [41].

## Evidence for the Hypothesis: The Neuroanatomy of AD

Our understanding of AD neurobiology has been transformed by a substantial body of neuroimaging data which demonstrates that the neurodegenerative process has a predictable and regionally specific evolution in the brain [1]. The very earliest disease targets in AD remain somewhat contentious but are likely to include entorhinal cortex and brainstem sites, including locus coeruleus, dorsal raphe nuclei or nucleus tractus solitarius [2,8,84]. Consistent with the early disruption of circadian and sleep physiology observed clinically, neuropathological evidence in AD clearly implicates ascending brainstem pathways that govern the sleep-wake cycle at or near the outset of the disease, including cholinergic, dopaminergic, melatoninergic and neurosteroid systems [2,4,27,42,56,63]. AD is a major cholinergic deficiency state [66], and cell loss in the nucleus basalis is correlated with the magnitude of cholinergic depletion in cerebral cortex [73]. In addition, neuronal counts in the locus coeruleus are reduced by some 80% in AD [57], with marked atrophy of the suprachiasmatic nucleus [67]. Involvement of the nucleus tractus solitarius could have particularly potent effects, as this area serves as a major ‘signalling hub’, integrating brainstem functions with the circadian pacemaker and widespread forebrain projection zones [84].

The forebrain projection targets of the ascending pathways disrupted in AD are widely distributed in the cerebral cortex, thalamus and other subcortical structures. However, there is a precise segregation of targets according to the specific transmitter systems involved [73]. For example, in the case of the cholinergic system, the hippocampus, amygdala and paralimbic structures receive particularly dense cholinergic innervation, and subregional variations in the strength of cholinergic inputs are evident within these structures. Furthermore, cholinergic projections to particular structures originate in distinct cholinergic neuronal pools in the brainstem and basal forebrain. This stratified organisation would support the mnestic functions mediated by the cholinergic system in the healthy brain and suggests a general mechanism for ‘gating’ sensory information to the limbic system [73]. In pathophysiological terms, such anatomical differentiation provides a potential substrate for the regionally directed spread of pathology in AD, effectively ‘focusing’ the neurodegenerative process on vulnerable zones in the DMN and potentially establishing a gradient of regional vulnerability across the network (see fig. 1).

Early involvement of brainstem neurotransmitter systems may therefore explain the integral vulnerability of the DMN in AD [6,8,12,53]. Neurotransmitter cycling is closely linked to neural activity [54], suggesting that altered patterns of neural activity in the DMN projection zones of damaged neurotransmitter pathways may govern the expression of AD. The evidence we have presented above demonstrates that sleep-wake cycling is a particularly potent example of large-scale alterations in neural activity focused in the DMN.

## Evidence for the Hypothesis: Cellular and Molecular Links between Sleep and Neurodegeneration

Previous animal work has suggested that altered patterns of sleep-wake activity may directly promote neurodegenerative brain damage and these effects could operate early in the pathophysiological cascade. Increased apoptosis, cytoskeletal disintegration and neuronal loss have been observed in the locus coeruleus of rat brain after REM sleep deprivation [44]. Neurons in the dorsal raphe nucleus may increase in size after REM sleep deprivation as a partial compensatory response to injury and (if not reversed) a potential prerequisite for neurodegeneration [44]. Cell size can be normalised by prazosin, suggesting that the response is mediated by noradrenergic pathways [44].

At a molecular level, potential links between sleep-related activity and substrates for neurodegenerative damage have been identified. Disrupted circadian function in mouse models with reduced expression of clock gene proteins (key regulators of circadian rhythm) is associated with accelerated ageing and impaired cognition via sleep-dependent and sleep-independent effects [27]. Sleep duration has been shown to be linearly related to telomere length in men, suggesting that in human subjects chronic sleep deprivation may also accelerate ageing [85]. In rat cerebral cortex, chronic sleep deprivation has a unique molecular profile of gene upregulation compared with shorter-term sleep deprivation [22]. More specifically, genetic factors may link sleep disruption with the pathophysiology of AD. While the effect of apolipoprotein E status has been inconsistent [58,67], both increased susceptibility to sleep disturbance in AD [58] and overall risk of developing AD [86] have been linked to polymorphisms of the gene-coding monoamine oxidase A, which helps govern the availability of serotonin in melatonin synthesis. This genetic factor also provides a potential link to mood disturbance, in line with evidence that depressive episodes can predict subsequent development of AD [40].

At a cellular and synaptic level, how might sleep disturbance promote AD-associated pathophysiological alterations and, ultimately, neurodegeneration? Several general candidate mechanisms have been identified. These mechanisms interact and can be broadly considered as pro-inflammatory, pro-fibrillogenic and miscommunicative.

The changes in sleep circuitry and associated neurotransmitters in AD have pro-inflammatory effects. This pro-inflammatory state could have reciprocal pathogenic interactions with specific brainstem hub regions [84]. Sleep disruption is associated with dysregulation of the physiological stress response with altered secretion of cortisol and other neurosteroids, and these substances in turn modulate cholinergic functions [27,42]. In addition, sleep disruption leads to reduced cellular tolerance of oxidation stress, which in turn is likely to reflect loss of neuroprotective functions, increased generation of reactive oxygen species, altered transduction of intracellular metabolic signals and reduced DNA repair [27,87]. The outcome of this cascade is neuronal dysfunction and death. The cascade may be triggered by an AD-associated reduction in the release of melatonin [50]. A pro-inflammatory mechanism of cell death may operate in various disease processes (it may, for example, account for the finding of accelerated hippocampal atrophy in obstructive sleep apnoea, perhaps mediated by phasic hypoxia) [88,89]. AD specificity would, however, emerge both in the initial sequence of cellular events that triggers the pro-inflammatory cascade and in the anatomical configuration of the pathways that are primarily damaged by the cascade (see fig. 1).

Sleep disruption in AD is likely to promote deposition of pathogenic fibrillar proteins. Synaptic activity has been shown using in vivo microdialysis to regulate levels of β-amyloid in interstitial fluid [45]. More specifically, in transgenic APP mice, sleep deprivation is associated with increased levels of interstitial fluid β-amyloid and, subsequently, increased deposition of amyloid plaques, while sleep induction with an orexin antagonist decreases plaque burden relative to control mice [26]. In human subjects, the diurnal level of β-amyloid-42 (the soluble oligomeric species implicated in generating amyloid plaques) in cerebrospinal fluid (CSF) is increased by wakefulness and decreased by sleep [24], while the level of β-amyloid in interstitial fluid is regulated by endogenous neuronal activity (as indexed by lactate production) [45]. Manipulation of synaptic vesicle release affects β-amyloid levels in interstitial fluid, suggesting a direct mechanistic link [45]. Conversely, both the diurnal fluctuation of CSF β-amyloid levels and the sleep-wake cycle can be normalised by immunisation against β-amyloid-42 in transgenic mouse models of AD [33]. Melatonin has anti-fibrillary actions [31] and inhibits deposition of β-amyloid and development of cognitive deficits in mouse models of AD [23,27,28]. Although clinical trials of melatonin have had somewhat mixed results [90,91,92], there is some evidence that it can delay deposition of β-amyloid in mouse models, improve survival in mouse models of AD [28], and improve cognition in patients with mild cognitive impairment [38]. Effects of sleep-wake alterations on pathogenic protein deposition are not restricted to β-amyloid. A similar link has been demonstrated for protein tau, the other major culprit protein implicated in AD pathogenesis. In mice, tau content is increased in the pons, preoptic area and hippocampus after 24 h of REM sleep deprivation and in the frontal cortex after 72 h of REM sleep deprivation [32]. These findings suggest that sleep disruption may lead to tissue-specific alteration in the neural cytoskeleton associated with protein tau deposition. In murine models of sleep deprivation there is evidence of an adaptive response to accumulated misfolded proteins which involves upregulation of unfolded protein response mechanisms in the endoplasmic reticulum. This adaptive response is impaired in aged mice, leading to accumulation of misfolded proteins and accelerating the proapoptotic cascade [30].

A further possible mechanism that could link sleep disruption to neurodegeneration is alteration in the pattern of synaptic activity, leading to neuronal miscommunication [10]. This is presently the least well worked out of the candidate mechanisms we consider, but might potentially precede and instigate pro-inflammatory and pro-fibrillary cascades. Furthermore, it provides a potential modus operandi for the trans-synaptic spread of pathology across the vulnerable network, an emerging principle that is likely to be relevant to the evolution of a range of neurodegenerative diseases [9]. We propose that neuronal miscommunication across synapses may be the fundamental mechanism underpinning the hypnic hypothesis of AD, leading processes of frank degeneration. Cellular miscommunication need not be restricted to neurons; it might also blight neuronal-glial interactions [10]. Abnormal or inefficient synaptic activity could restrict delivery of trophic factors or promote intercellular transfer of misfolded pathogenic proteins [10]. Diffusive or ‘prion-like’ spread of pathogenic proteins has been demonstrated in animal models for both β-amyloid [16] and tau [15]. In addition, altered synaptic activity might itself promote local protein misfolding in the presence of pre-toxic substrates (e.g. enhanced tau phosphorylation via increased nerve growth factor release [4]) or might exert less specific excitotoxic effects (e.g. by triggering the pro-inflammatory cascade). Once present, pathogenic proteins may further degrade synaptic function. For example, β-amyloid fragments may alter synaptic transmission via an AMPA-mediated glutamatergic mechanism [93], while tau modulates the synchronous firing of neurones [94].

The mechanisms we have considered would have many potential points of convergence and amplification (see fig. 1). For example, melatonin, in addition to its neuroprotective and anti-fibrillary effects, enhances cholinergic function [95]. Melatonin deficiency would therefore tend to promote abnormal activity at cholinergic synapses. Increased neuronal activity may promote accumulation of β-amyloid [7], while deposition of β-amyloid in turn disrupts synaptic function [6,96]. The pro-inflammatory cascade may reduce clearance of pathogenic proteins, thereby accelerating fibrillary transformation, and may also inhibit synaptic repair [10]. Once the neurodegenerative process in AD becomes established, the pro-oxidant state would be perpetuated by the deposition of amyloid plaques and neurofibrillary tangles. Pathogenic proteins may act to consolidate and perpetuate the abnormal toxic state (e.g. protein tau itself may indirectly regulate the sleep-wake cycle) [94].

## Testing the Hypothesis

We propose this hypnic hypothesis of AD with caveats. Despite recent progress, our understanding of sleep neurobiology and AD pathophysiology remains limited. In particular, the mechanisms that link molecular and cellular events to neural circuit damage remain to be established in detail. We do not, of course, propose that sleep disturbance is the sole factor driving the development of AD – rather, we envisage it as a key factor in consolidating and amplifying the neurodegenerative process in core vulnerable brain areas. Taking these caveats into account, the hypnic hypothesis makes certain specific predictions with potentially far-reaching implications that might be tested in future work (summarised in table 1). The hypothesis implies, for example, that chronic sleep disruption might promote the development of AD and, conversely, that appropriate treatment to regularise sleep patterns, in particular to augment REM sleep, might prevent (or at least retard) its development. Arguably, any hypnic effect might be relatively more important early in the evolution if AD, before the neurodegenerative process is fully established and secondary pathogenic mechanisms have come into play. Aside from its importance in establishing the pathophysiological sequence, early-stage disease is a more feasible candidate for detailed polysomnographic studies than established dementia.

Perhaps the most basic requirement will be a detailed, prospective longitudinal analysis to track sleep physiology in relation to early markers of disease onset (ideally, predating the development of cognitive decline). This would be logistically challenging but could be directed to cohorts at known risk of developing AD (e.g. carriers of disease-causing mutations), capitalising on recent progress in identifying novel biomarkers of AD such as CSF tau, β-amyloid assays and amyloid brain imaging, as well as laboratory markers of circadian function (such as melatonin). There is a particular need to investigate REM sleep and dreaming in AD, about which information remains very limited. Complementary approaches should include large-scale clinical epidemiological studies to assess relative AD risk associated with habitual sleep patterns (e.g. those associated with particular occupations) and, potentially, therapeutic trials of sleep-regularising agents, if these can be administered sufficiently early in the course of disease. Ultimately, detailed pathological correlation will be required in individuals with AD undergoing comprehensive assessment of sleep physiology during life.

More fundamentally, the pathophysiology of sleep remains to be worked out in detail at cellular and circuit levels. This will entail the development of animal and in vitro models that can capture the effects of AD pathology, the effects of perturbed cellular interactions on the spread of AD pathology in model neural circuits, and compensatory cellular and synaptic responses. Animal models also allow evaluation of the impact of specific manipulations of sleep physiology (e.g. selective REM deprivation or augmentation) on molecular and behavioural phenotypes. In addition, more information is needed about early markers of DMN pathophysiology. This might entail, for example, an analysis of complex behavioural alterations (potentially expressed in social, affective or mood changes) that are not conventionally regarded as part of the typical phenotype of established AD but which may well go under-recognised [40]. Animal models are potentially particularly valuable for charting the evolution of circadian disturbances in relation to neurodegenerative pathology and the effects of interventions [25].

Finally, we speculate that the hypnic hypothesis of AD might exemplify a much broader class of disease mechanisms, namely, neurodegeneration instigated by abnormal neural network behaviour. Sleep illustrates a gross and pervasive alteration in brain function. It remains to be established whether more subtle, habitual patterns of brain activity might promote other kinds of pathophysiological deterioration. Examples might include the cognitive patterns associated with certain occupations, which have been associated epidemiologically with particular neurodegenerative syndromes [97], or the role of educational attainment [54]. Consistent with this notion, sensorially enriched environments have been shown to affect the development of the neurodegenerative process (as indexed by rates of deposition of amyloid plaques) in animal models of AD [98,99,100]. These effects may be mediated in part by exercise-associated influences on cholinergic activity and neuronal plasticity [101]. If we are to explain the syndromic specificity of particular neurodegenerative pathologies, it is critical that we uncover the processes that link molecular lesions to large-scale network disintegration [9]. The functional properties of neural circuits and their modes of operation in generating complex (including circadian) behaviours are a largely unexplored but potentially critical link by which cellular events might imprint and propagate themselves across vulnerable networks at the level of the whole brain. The creation of programmable neural networks in vivo offers the exciting prospect of assessing such processes directly [102].

## Figures and Tables

**Table 1:** Key features, predictions and proposed tests of the hypnic hypothesis of AD

Features	Predictions	Tests
*Systems level*		
– Pathology in brainstem pathways critical for sleep-wake and circadian physiology – Pathological changes in brainstem nuclei implicated in circadian control – Dysfunction and degeneration of ascending neurotransmitter pathways – Altered sleep/circadian physiology	– Histopathological analyses in presymptomatic subjects – Functional, tractographic studies of brainstem pathways – Longitudinal sleep analysis in presymptomatic subjects – Animal models (especially transgenic)

– Sleep disruption drives subsequent neurodegeneration	– Disrupted sleep/circadian patterns early in clinical course precedes cognitive decline, cortical dysfunction and atrophy	– Longitudinal sleep analyses in presymptomatic subjects with parallel neuroimaging – Early intervention to ameliorate sleep disruption – Epidemiological studies assessing effects of premorbid neuronal activity (e.g. occupation, educational attainment) – Animal models (especially transgenic)

– DMN neurodegeneration linked with sleep disruption	– Specific DMN dysfunction, disintegration correlated with sleep/brainstem indices	– Functional, structural neuroimaging of DMN against behavioural indices in relation to sleep analyses

– REM sleep is an active ‘rescue’ state	– REM deprivation and augmentation effects on cognitive/neuronal function	– Sleep analyses in AD subjects – Animal models with selective REM deprivation

– Self-amplification of sleep disruption effects	– Neurodegeneration and sleep alteration accelerating in tandem	– Longitudinal sleep analyses, cognitive tests, neuroimaging in AD subjects – Sleep parameters manipulated in animal models

*Cellular level*		
– Sleep disruption drives pro-inflammatory and oxidant states	– Pro-inflammatory, pro-oxidant responses correlated with circadian indices	– Animal models with biochemical studies – Human plasma studies

– Sleep disruption drives protein misfolding and accumulation	– β-amyloid, tau levels correlated with circadian indices	– Interstitial and CSF β-amyloid and tau levels – Pathological studies with regional quantitation

– Cellular miscommunication drives neurodegeneration	– Persistent patterns of altered synaptic activity drives neurodegeneration	– Manipulate environment and neurochemistry (animal models) – Synthetic neural networks modelling sleep stage activity

*Molecular level*		
– Altered expression of circadian genes predisposes to neurodegeneration	– Altered expression profiles of (e.g. circadian clock) gene effects on neurodegeneration	– Genomic, endophenotypic analyses – Transgenic animal models

– AD-related genes produce circadian alterations	– Primary alterations in circadian indices in at-risk individuals	– Sleep analyses in at-risk young subjects
